# Distinct and Overlapping Requirements for Cyclins A, B, and B3 in *Drosophila* Female Meiosis

**DOI:** 10.1534/g3.116.033050

**Published:** 2016-09-20

**Authors:** Mohammed Bourouh, Rajdeep Dhaliwal, Ketki Rana, Sucheta Sinha, Zhihao Guo, Andrew Swan

**Affiliations:** Department of Biological Sciences, University of Windsor, Ontario N9B 3P4, Canada

**Keywords:** cyclin, Cdk1, meiosis, *Drosophila*

## Abstract

Meiosis, like mitosis, depends on the activity of the cyclin dependent kinase Cdk1 and its cyclin partners. Here, we examine the specific requirements for the three mitotic cyclins, A, B, and B3 in meiosis of *Drosophila melanogaster*. We find that all three cyclins contribute redundantly to nuclear envelope breakdown, though cyclin A appears to make the most important individual contribution. Cyclin A is also required for biorientation of homologs in meiosis I. Cyclin B3, as previously reported, is required for anaphase progression in meiosis I and in meiosis II. We find that it also plays a redundant role, with cyclin A, in preventing DNA replication during meiosis. Cyclin B is required for maintenance of the metaphase I arrest in mature oocytes, for spindle organization, and for timely progression through the second meiotic division. It is also essential for polar body formation at the completion of meiosis. With the exception of its redundant role in meiotic maturation, cyclin B appears to function independently of cyclins A and B3 through most of meiosis. We conclude that the three mitotic cyclin-Cdk complexes have distinct and overlapping functions in *Drosophila* female meiosis.

The cyclin dependent kinases (Cdks) and their cyclin partners are the core regulators of the eukaryotic cell cycle, and are responsible for the phosphorylation of multiple target proteins at each phase of the cell cycle. Genetic studies have revealed that cyclins have considerable redundancy. For example, in budding yeast, Clbs1–4 are active in mitosis but none are essential, and one of them (Clb2) is sufficient to drive mitosis in the absence of the other three (Richardson *et al.* 1992). Meanwhile, in fission yeast, a single cyclin (Cdc13) is sufficient to carry out a complete cell cycle (Fisher and Nurse 1996).

In higher eukaryotes, Cdk1 pairs with cyclins of three related types: cyclin A, (CycA), cyclin B (CycB), and cyclin B3 (CycB3) (reviewed in Fung and Poon 2005; Nieduszynski 2002). Vertebrates including human, mouse, and *Xenopus laevis*, have two *CycA* genes (*A1* and *A2*), two *CycB* genes (*B1* and *B2*), and a single *CycB3* gene. Both *CycB1* and *CycA2* are required for mitosis in somatic cells (Furuno *et al.* 1999), while *CycA1* and *CycB2* are expressed at low levels in somatic cells and are not required for mitosis. *CycB3* is also either not expressed or expressed at very low levels in somatic cells in vertebrates (Nguyen *et al.* 2002).

In *Drosophila melanogaster*, which has a single representative of each mitotic cyclin type, the redundancies among cyclins again differ. *CycB* is not essential for viability in *Drosophila*, though homozygous mutants display some spindle abnormalities and cell cycle timing defects (Knoblich and Lehner 1993; Jacobs *et al.* 1998). Unlike mammalian *CycB3*, *Drosophila CycB3* is expressed in mitotic tissues. It is not required for viability but *CycB,CycB3* double mutants are embryonic lethal, indicating that it plays an essential redundant role with *CycB* (Jacobs *et al.* 1998). *CycA* is the only essential mitotic cyclin in *Drosophila*. This appears to reflect a requirement in G2 to inhibit the activity of the APC/C and thus allow itself, CycB, and CycB3 (and other mitotic regulators) to accumulate and drive mitotic entry (Reber *et al.* 2006). Outside of this requirement, CycA functions redundantly with CycB and CycB3 (Knoblich and Lehner 1993; Jacobs *et al.* 1998). In the syncytial divisions of the early embryo, the three mitotic cyclins again appear to play largely overlapping roles. RNAi knockdown reveals CycB to be the primary mediator of nuclear envelope breakdown (NEB), though the other two cyclins also contribute (McCleland and O’Farrell 2008; McCleland *et al.* 2009). CycA and CycB are required together to achieve a proper metaphase. CycB3 appears to be the major driver of anaphase, and may function in this respect by promoting APC/C activity (McCleland *et al.* 2009; Yuan and O’Farrell 2015), a function that appears to be conserved (Deyter *et al.* 2011; Zhang *et al.* 2015). Therefore, the cyclins are collectively essential for mitosis but they show considerable overlap in their individual contributions.

The roles of the three mitotic cyclins in *Drosophila* meiosis are not as clear. To date, only *CycB3* has been studied, and was found to be necessary for the completion of meiosis following ovulation (Jacobs *et al.* 1998). *CycB* is essential for fertility, and in females this is due to a requirement in the mitotic divisions that precede meiosis (Jacobs *et al.* 1998; Wang and Lin 2005). This premeiotic requirement has so far precluded the study of *CycB* in meiosis. The role of *CycA* in meiosis is also not known, due to the fact that null mutants are lethal (Lehner and O’Farrell 1989). In this paper, we use conditional RNAi knockdown and mutants to determine the function of CycA, CycB, and CycB3 in meiosis.

*Drosophila* female meiosis is initiated early in oogenesis and is completed shortly after ovulation (see McKim *et al.* 2002 and Von Stetina and Orr-Weaver 2011 for reviews of meiosis). The oocyte arises within a cluster of 16 interconnected germline cells that forms in the germarium. The remaining 15 germline cells differentiate as nurse cells that provide nutrients to the growing oocyte. The oocyte with associated nurse cells and surrounding somatic follicle cells form an egg chamber that pinches off from the germarium and progresses through 14 stages that can be distinguished by morphological features (see Spradling 1993 for a review of oogenesis). During the period of oocyte growth, from stages 1–12 of oogenesis, the oocyte remains in meiotic prophase. For most of this period, the oocyte nucleus is transcriptionally silent and the chromatin is compacted into a structure called the karyosome. Meiotic maturation, marked by NEB, occurs in midstage 13. There are no centrosomes in the oocyte and microtubules organize around the already congressed meiotic chromosomes to form the meiosis I spindle. The stage 14 oocyte (also referred to as a mature oocyte) is arrested in metaphase I and remains so until ovulation triggers anaphase I (see Foe *et al.* 1993 or Page and Orr-Weaver 1997 for reviews of meiotic events that follow ovulation). Anaphase I is followed immediately by entry into and progression through meiosis II. The completion of meiosis occurs within 20 min of ovulation, and is marked by the presence of four haploid nuclei in the dorsal anterior of the egg. The egg is fertilized as it passes through the oviduct, and a sperm-derived microtubule aster appears to guide one of the four maternal nuclei toward the male pronucleus. The male and female pronuclei together enter the first nuclear (syncytial) division of embryogenesis. Concurrent with zygote formation, the remaining three polar body nuclei come together, undergo NEB, and arrest with condensed chromatin arranged in a rosette pattern on a microtubule array. This polar body remains in the dorsal anterior of the egg until late in the syncytial divisions.

## Materials and Methods

### Drosophila stocks and crosses

*CycA^GLV21059^*, *CycB^HMS01015^*, *CycB^HMS01896^*, and *Cdk1^GL00262^* were obtained from the Bloomington *Drosophila* Stock Center (BDSC). *CycB^HMS01015^* recognizes a sequence between 551 and 571 on the *CycB-RB* transcript, while *CycB^HMS01896^* recognizes a sequence between 244 and 264. *Cyclin A^3′RNAi^* was generated using the valium 22 vector (obtained from TRiP, Harvard University) and recognizes a sequence between 2145 and 2165 in the *CycA-RC* transcript. *CycB^2^* is a null allele of *CycB* and for all experiments was used over *Df(2R)59A-B*, a deficiency that removes the *CycB* gene. *hs-CycB* (Knoblich and Lehner 1993) (one copy of a third chromosome insertion) was used to rescue the premeiotic defects of these null mutants. CycB expression from *hs-CycB* was induced by constant rearing at 29°. Under such conditions, *CycB^2^/Df(2R)59A-B;hs-CycB/+ (CycB^−/−^;hs-CycB)* females laid eggs for at least 2 wk, indicating rescue of the premeiotic defects. In most experiments, *CyO/CycB^2^(or Df(2R)59A-B)*; *hs-CycB/+* siblings of the *CycB^−/−^*;*hs-CycB* flies were used as controls. *CycB^2^*, *Df(2R)59A-B* and the *hs-CycB* transgenic stock were obtained from the BDSC. In other experiments, *yw* flies were used as wild-type controls. Unfertilized eggs were obtained by crossing virgin females to XO males (generated by crossing wild-type females to males carrying an attached X^Y chromosome). The Lamin C-GFP gene trap (*LamC^G00158^*) was obtained from the BDSC.

### Immunostaining and FISH

Eggs were collected at the stated intervals from apple juice agar plates in egg lay chambers. Eggs were dechorionated in bleach, and fixed and devitelinized simultaneously by shaking in heptane/methanol. Following rehydration, eggs were processed for immunofluorescence by standard methods. To obtain late stage oocytes for FISH or DNA staining, ovaries were dissected in Isolation Buffer (55 mM NaOAc, 40 mM KOAc, 110 mM sucrose, 1.2 mM MgCl_2_, 1 mM CaCl_2_, and 100 mM HEPES, pH 7.4) with collagenase to dissociate individual oocytes. Oocytes were fixed in 50:50 heptane: 3.7% formaldehyde in PBS, 0.2% Tween 20. For immunostaining of stage 14 oocytes, ovaries were fixed as above, then transferred to 100% methanol and sonicated to remove vitelline membranes. Oocytes were then rehydrated and an extraction step was performed with 1:1 octane:PBS, 0.5% Triton-X for 30 min, then processed for immunostaining by standard methods. Rat anti-Tubulin YL1/2 (Sigma) was used at 1/500, mouse anti-Lamin ADL195 (Developmental Studies Hybridoma Bank) was used at 1/100. DNA was labeled with mouse anti-Histone H3 (Chemicon) used at 1/1000, or with the DNA dye Oligreen (Invitrogen) or propidium iodide (Invitrogen). Alexa dye conjugated secondary antibodies (Invitrogen) were used at 1/1000. FISH was performed on Methanol-fixed eggs or ovaries as described (Dernburg 2000), using a probe against the 359 bp pericentromeric repeat on the X-chromosome.

### Western blotting

Extracts for western blotting were obtained from ovaries that were dissociated into individual eggs and egg chambers by treatment with collagenase. Stage 13 and stage 14 eggs were selectively enriched by repeated rounds of mixing and then pouring off the slower settling smaller egg chambers. Laid eggs were obtained from 0–2 hr collections from females fertilized by XO males. Mouse anti-CycB and mouse anti-CycA antibodies (Developmental Studies Hybridoma Bank) were used at 1/20 and 1/5, respectively. Rabbit anti PSTAIR antisera (Santa Cruz) was used at 1/1000 and mouse anti-Actin (Millipore) was used at 1/5000. HRP-conjugated secondary antibodies (Roche) were used at 1/7000 and detected by ECL.

### Data availability

All *Drosophila* strains and DNA constructs generated in this study are available upon request. The authors state that all data necessary for confirming the conclusions presented in the article are represented fully within the article.

## Results

### Knockdown of the mitotic cyclins in female meiosis

**Figure 1:**
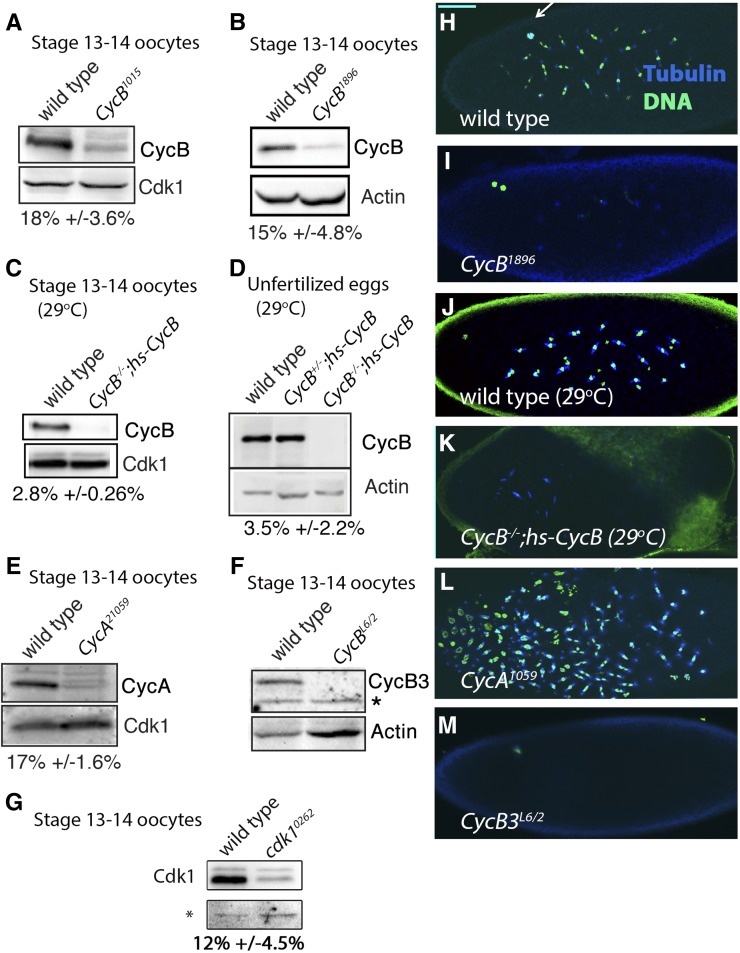
Characterization of cyclin and *Cdk1* knockdowns. (A–G) Western blots on oocyte stage 13 and 14 oocytes (see *Materials and Methods*) and unfertilized eggs from wild-type (*yw*) and cyclin knockdown females, probed for Cyclins A, B, B3, and Cdk1 (PSTAIR antibody) as indicated. In (A), (B), (E), and (G) the *UAS-RNAi* line is driven by *mat-Gal4*. Percentages below each blot represent percent of wild-type levels in the knockdown or mutant, based on densitometry from three experiments ± SEM. Either Cdk1 or Actin is used as a loading control, except in (G) where a nonspecific band that correlates with total protein levels is used. For *CycB3^L6/2^* (F), no CycB3 protein could be detected. The * in (F) and (G) indicates a nonspecific band. (H–M) Embryos labeled for Tubulin and DNA as indicated in (H). In (I) and (L) the *UAS-RNAi* line is driven by *mat-Gal4*. The wild-type embryos in (H) and (J) are undergoing syncytial divisions. Arrow in (H) points to the polar body. (I) *CycB^1896^* embryo undergoing aberrant syncytial divisions. Blue foci are centrosomes, most of which are not associated with chromatin. The two chromatin masses in the dorsal anterior (top left) are the meiotic products. (J) and (K) Wild-type (*yw*) and *CycB^−/−^;hs-CycB* embryos both raised at 29°. Development appears normal in wild-type at this temperature (J). In (K), mitotic spindles appear disorganized and clustered near the anterior of the egg. It is not possible to clearly distinguish the meiotic products in this egg. (L) Embryo from *CycA^21059^* undergoing aberrant syncytial divisions. (M) Embryo from *CycB3^L6^*^/2^ containing a single meiotic spindle, indicative of a meiosis I arrest. Scale bar in H = 50 μm and applies to (H–M). Cdk, cyclin-dependent kinase; Cyc, cyclin; RNAi, RNA interference.

To study the requirements for the mitotic cyclins in meiosis, we used a combination of germline-specific RNAi knockdown (for *CycA* and *CycB*) and mutants (for *CycB* and *CycB3*) (Figure 1). To deplete CycB in the female germline we expressed either of two nonoverlapping *UAS-RNAi* lines, *CycB^HMS01896^* and *CycB^HMS01015^* (hereafter *CycB^1896^* and *CycB^1015^*) under the control of the *maternal α-Tubulin-Gal4-67 (mat-Gal4)* driver (Staller *et al.* 2013). This *Gal4* driver induces *UAS* transgene expression after the premeiotic divisions and remains on throughout oogenesis (Staller *et al.* 2013). We will refer to oocytes, eggs, or embryos derived from females that express these RNAi lines under *mat-Gal4* control simply as *CycB^1896^* and *CycB^1015^* oocytes, eggs, or embryos. The same terminology will be used to describe other *mat-Gal4*-mediated gene knockdowns in this paper. Western blots on extracts enriched for stage 13 and stage 14 oocytes (see *Materials and Methods*) indicate that *CycB^1015^* and *CycB^1896^* oocytes have 18 and 15% of wild-type CycB protein levels, respectively (Figure 1, A and B). Egg production from *CycB^1015^* and *CycB^1896^* females is similar to wild-type, indicating that the premeiotic function of CycB (Wang and Lin 2005) is not affected. However, both *CycB^1015^* and *CycB^1896^* eggs fail to hatch and appear to arrest in the syncytial embryonic cycles (Figure 1I, compare to Figure 1H). As an independent approach to depleting CycB, we generated a conditional mutant allele by placing a heat shock-inducible *CycB* transgene (*hs-CycB*) (Knoblich and Lehner 1993) into a *CycB*-null background (*CycB^2^/Df(2R)59A-B* – hereafter referred to as *CycB^−/−^*). When *CycB^−/−^*;*hs-CycB* flies are kept at 29°, CycB protein levels are reduced to 2.8% of wild-type levels in stage 13–14 oocytes and 3.5% of wild-type levels in unfertilized eggs (Figure 1, C and D). Eggs from these *CycB^−/−^*;*hs-CycB* females arrest in the syncytial cycles, similar to the two RNAi lines (Figure 1K). When either wild-type females or females carrying *hs-CycB* in a *CycB* heterozygous background (*CycB^+/−^*;*hs-CycB*) are raised at 29°, their eggs have normal CycB levels, develop normally, and hatch (Figure 1, D and J and data not shown). Therefore, the lethality of *CycB^−/−^*;*hs-CycB* embryos is due to the loss of *CycB* and not due to either elevated temperature or the expression of *hs-CycB*. In subsequent experiments to be described below, we found that the two RNAi lines and *CycB^−/−^*;*hs-CycB* produce similar meiosis phenotypes, arguing that the knockdowns are specific. Unfortunately, *CycB^−/−^*;*hs-CycB* is lethal when combined with *CycB3* mutants, precluding its use in double or triple knockdowns with *CycB3* (data not shown). *CycB^−/−^*;*hs-CycB* females also produce far fewer eggs than the RNAi lines (data not shown). For these reasons, we relied on the RNAi lines for most experiments.

To deplete CycA during female meiosis, we crossed *mat-Gal4* to a *UAS-RNAi* line that targets *CycA*, *CycA^GLV21059^* (*CycA^21059^*). CycA protein levels are reduced to 17% of wild-type levels in stage 13–14 oocytes from *CycA^21059^* females (Figure 1E). These females produce eggs that fail to hatch. Embryos go through several aberrant syncytial divisions and typically arrest prior to cellularization (Figure 1L). To verify the phenotypes from this RNAi line, we first attempted to generate a conditional *CycA* mutant analogous to *CycB^−/−^*;*hs-CycB*. However, we were unable to rescue the zygotic lethality of a *CycA* null mutant using a *hs-CycA* transgene (Knoblich and Lehner 1993) (data not shown). Therefore, we generated a second, nonoverlapping RNAi line, *CycA^3′RNAi^*. Unfortunately, this line produces a significantly weaker phenotype than *CycA^21059^*: approximately half of the eggs produced by *CycA^3′RNAi^* females hatch. Nonetheless, as described later (Figure 9), this line and *CycA^21059^* produce the same phenotype in combination with *CycB3* mutations. We conclude that *CycA^21059^* and *CycA^3′RNAi^* both produce a specific knockdown of *CycA* in female meiosis.

Unlike *CycB* and *CycA*, *CycB3* is not required for viability or for the germline mitotic divisions that precede oocyte formation, and thus its role in meiosis can be directly assessed using mutant alleles (Jacobs *et al.* 1998; Yuan and O’Farrell 2015). In this study, we use flies transheterozygous for the strong alleles, *CycB3^L6540^ (CycB3^L6^)* and *CycB3^2^* (Jacobs *et al.* 1998). Stage 13–14 oocytes from *CycB3^L6/2^* females have no detectable protein by western blot (Figure 1F) and, as previously described (Jacobs *et al.* 1998), fertilized eggs from *CycB3^L6/2^* females fail to develop (Figure 1M).

### Meiotic maturation in Drosophila females requires Cdk1 and any mitotic cyclin

**Figure 2:**
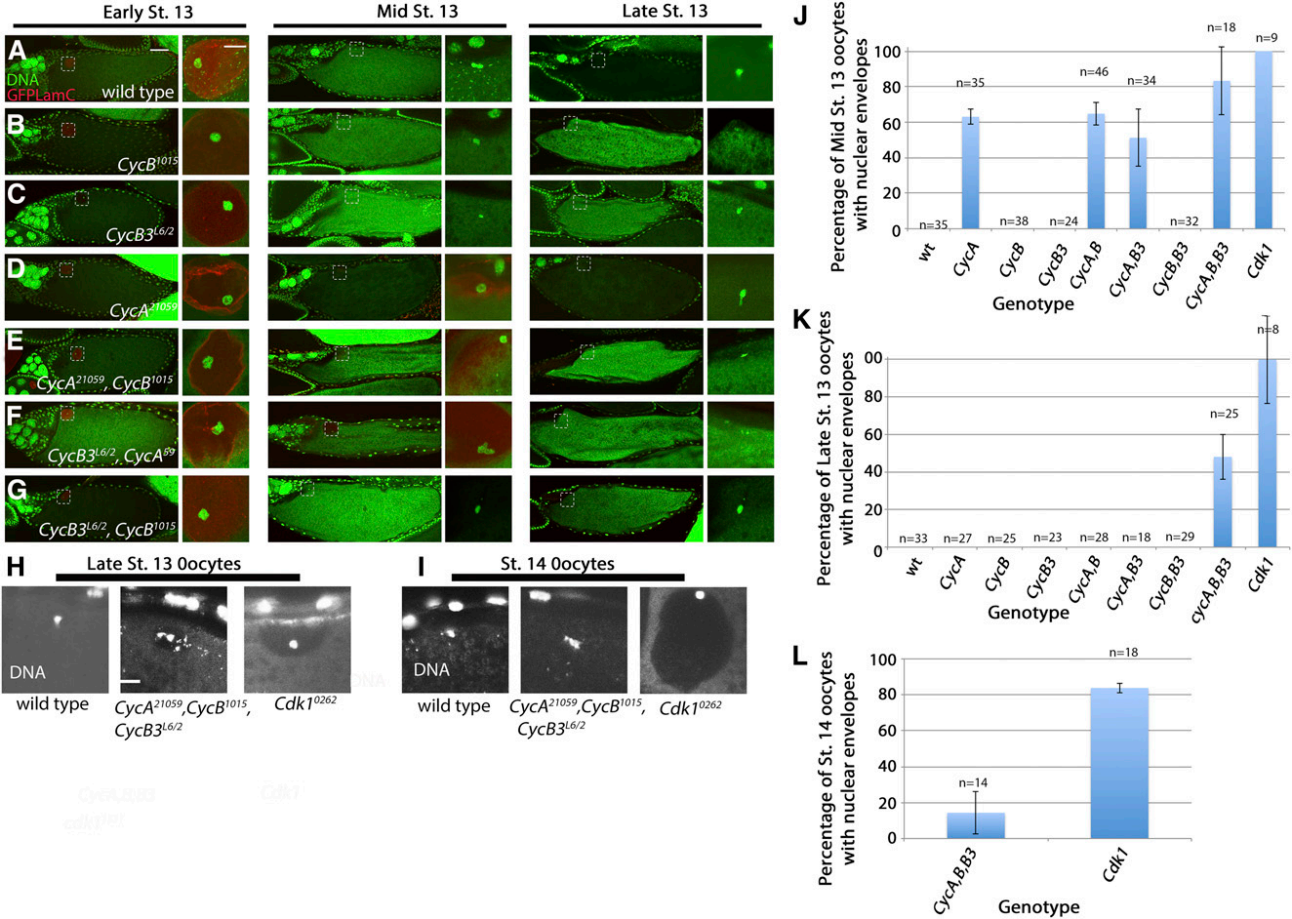
Mitotic cyclins play redundant roles in meiotic maturation. *UAS-RNAi* lines *CycA^21059^*, *CycB^1015^*, and *Cdk1^0262^* are all driven by *mat-Gal4*. (A–G) Early, mid, and late stage 13 oocytes labeled for GFP-Lamin C (red) and DNA (propidium iodide in green). For each stage, the first panel shows the full oocyte (scale bar = 50 μm). The dashed box indicates the site of the oocyte nucleus, and this is shown in higher magnification in the smaller panel to the right (scale bar = 10 μm). Staging is based on the appearance of nurse cell nuclei and dorsal appendages. Presence of a nuclear envelope is assessed from Lamin staining and from exclusion of propidium iodide signal from a region around the karyosome. (A) Oocytes from wild-type, showing presence of a nucleus in early stage 13 and absence in mid and late stage 13. The same timing of NEB is seen in *CycB^1015^* (B) and *CycB3^L6/2^* (C), as well as *CycB^1015^*,*CycB3^L6/2^* (G). (D) Oocytes from *CycA^21059^* showing presence of a nucleus in early and midstage 13 and absence in late stage 13. The same delayed NEB is seen in *CycA^21059^*,*CycB^1015^* (E) and *CycA^21059^*,*CycB3^L6/2^* (F). (H) and (I) Late stage 13 and stage 14 oocytes stained with Oligreen to detect DNA and presence of a nuclear envelope (by exclusion of Oligreen signal from a region around the karyosome). NEB had occurred in the wild type in (H) and (I). The late stage 13 *CycA^21059^*,*CycB^1015^*,*CycB3^L6/2^* oocyte in (H) shows partial clearing of cytoplasm near the bulk of chromatin, suggesting that NEB is incomplete. NEB is complete in the stage 14 *CycA^21059^*,*CycB^1015^*,*CycB3^L6/2^* oocyte in (I). A nuclear envelope is present in the late stage 13 and stage 14 *Cdk1^0262^* oocytes (H and I). (J–L) NEB timing in cyclin and *Cdk1* knockdown oocytes. Specific alleles are the same as those indicated for (A–I). Graphs represent percentage of oocytes with fully intact oocyte nuclei, as determined by GFP-Lamin C or DNA staining. Graphs are based on two or more independent experiments. Error bars indicate SEM. Cdk, cyclin-dependent kinase; Cyc, cyclin; GFP, green fluorescent protein; NEB, nuclear envelope breakdown; RNAi, RNA interference.

Cdk1-CycB is the critical mediator of meiotic maturation, or NEB, in diverse organisms (Labbe *et al.* 1989; Nebreda and Ferby 2000). Cdk1 is at least partially responsible for meiotic maturation in *Drosophila*: a temperature-sensitive *Cdk1* allele combination as well as mutations in the gene for the Cdk-activating phosphatase, *Cdc25/twine*, both result in a delay though not a complete failure of NEB (Von Stetina *et al.* 2008). The specific cyclin partner or partners for Cdk1 in meiotic maturation are not known for *Drosophila*. To determine if any of the three mitotic cyclins play a role in NEB, we examined NEB timing in single knockdown oocytes (Figure 2, A–D, J, and K). In oocytes labeled with the DNA dye propidium iodide, the presence of a nuclear envelope can be inferred from the appearance of a cleared area around the meiotic chromatin. We also used a *GFP-Lamin C* gene trap (Morin *et al.* 2001) to directly identify the nuclear envelope. In the wild type, a nucleus was detected in early stage 13 but not in midstage 13 oocytes, indicating that NEB occurs between early and midstage 13 (Von Stetina *et al.* 2008) (Figure 2, A and J). NEB timing was not altered in *CycB^1015^* (Figure 2, B and J). Even the stronger hypomorphic conditon, *CycB^−/−^*;*hs-CycB*, had no effect on the timing of NEB*:* 9/9 midstage 13 and 18/18 late stage 13 *CycB^−/−^*;*hs-CycB* oocytes that we examined had undergone NEB. *CycB3^L6/2^* oocytes also undergo NEB without any apparent delay (Figure 2, C and J). On the other hand, approximately 60% of mid stage 13 (or mid-stage 13) *CycA^21059^* oocytes still had a nucleus (Figure 2, D and J). By late stage 13, all *CycA^21059^* oocytes had undergone NEB (Figure 2, D and K). Therefore, *CycA^21059^* oocytes display a slight but consistent delay in NEB. *CycA^21059^* does not eliminate all CycA protein, and it is possible that a stronger knockdown would lead to a more pronounced NEB delay. No delay in NEB was observed in *CycA^3′RNAi^* oocytes (data not shown), presumably due to the weaker overall effectiveness of this RNAi line. Despite the caveat that we rely on a single RNAi line, these results suggest that CycA is necessary for timely NEB in female meiosis.

We next generated double knockdowns of the mitotic cyclins to determine if any of these combinations produce a further delay in NEB. Combination of *CycA^21059^* with either *CycB^1015^* or *CycB3^L6/2^* resulted in a delay in NEB timing, but this was not more severe than that of *CycA^21059^* alone (Figure 2, E, F, J, and K). Meanwhile, *CycB^1015^*,*CycB3^L6/2^* double knockdown oocytes appeared wild-type with respect to NEB timing (Figure 2, G, J, and K). We note that, in the specific case of *CycA^21059^*,*CycB^1015^*, the two *UAS-RNAi* lines might compete for limiting Gal4. This would result in reduced knockdown of both genes, potentially masking any synergy between the two knockdowns.

Finally, we determined the consequences of simultaneous knockdown of all three mitotic cyclins (by combining *mat-Gal4*, *CycA^21059^*,*CycB^1015^*, and the two mutant alleles of *CycB3*). *CycA^21059^*,*CycB^1015^*,*CycB3^L6/2^* oocytes displayed a more profound delay in NEB than the *CycA* single or double knockdown combinations. Of the late stage 13 oocytes from triple knockdown females, 48% still had nuclear envelopes (Figure 2, H and K). This compares to 0% in *CycA^21059^* alone and 0% in the *CycA,B* and *CycA,B3* double knockdown combinations (Figure 2K). Of stage 14 *CycA,B,B3* triple knockdown oocytes, 14% still had nuclear envelopes (Figure 2, I and L). Therefore, loss of all three mitotic cyclins can lead to a complete failure of NEB.

The NEB delay produced by knockdown of all three mitotic cyclins is stronger than that produced by loss of their common kinase partner Cdk1 (Von Stetina *et al.* 2008). This could reflect the hypomorphic nature of the *Cdk1* temperature-sensitive allele that was used previously. We obtained the RNAi line *Cdk1^GL00262^ (Cdk1^0262^)* (Ni *et al.* 2011) and expressed it under *mat-Gal4* control with the hope of obtaining a stronger phenotype. Western blotting revealed that this RNAi line reduces Cdk1 protein levels to 12% of wild-type levels (Figure 1G). All mid and late stage 13 oocytes from *Cdk1^0262^* had a nucleus (Figure 2, H, J, and K), while 83% of stage 14 oocytes still had an intact nucleus (Figure 2, I and L). Therefore, most oocytes fail to undergo NEB following knockdown of *Cdk1*.

### CycA is required for biorientation of homologs in meiosis I

**Figure 3:**
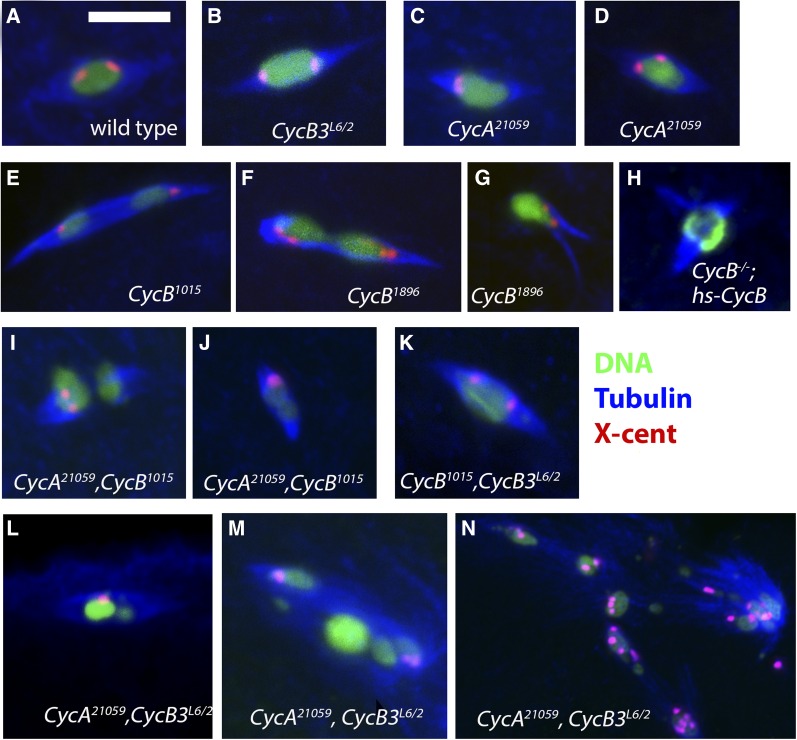
Requirement for mitotic cyclins in mature oocytes. Stage 14 oocytes labeled for DNA (Oligreen in green), Tubulin (blue), and X-cent FISH probe (red). Wild-type stage 14 oocytes (A) have a single compact mass of chromatin with two X-cent FISH dots oriented toward opposite poles of the spindle. (B) *CycB3^L6/2^* oocyte in a normal metaphase I arrest. (C, D) *CycA^21059^* oocytes displaying a failure of X-chromosome biorientation. Spindle morphology appears normal and chromatin appears as a single mass, but the X-cent FISH dots are not properly oriented toward opposite sides of the chromatin mass. (E and F) *CycB^1015^* and *CycB^1896^* oocytes in which chromatin is split into two masses. (G and H) *CycB^1896^* and *CycB^−/−^*;*hs-CycB* oocytes with aberrant meiosis I spindles. (I and J) *CycA^21059^*,*CycB^1015^* oocytes displaying a split chromatin phenotype (I) and biorientation defects (I and J). (K) *CycB^1015^*,*CycB3^L6/2^* oocyte in a normal metaphase I arrest. (L–N), *CycA^21059^,CycB3^L6/2^* double knockdown oocytes. The oocyte in (L) has a single chromatin mass that is not properly compacted, with a single X-cent FISH dot indicating failure of biorientation. The oocyte in (M) has multiple distinct chromatin masses and abnormal spindle. The oocyte in (N) has multiple chromatin masses and multiple X-cent FISH dots, indicative of rereplication. Scale bar in A =10 μm and applies to all panels. Cyc, cyclin; FISH, fluorescence *in situ* hybridization; wt, wild-type; X-cent, probe specific to the pericentric region of the X-chromosome.

**Figure 4:**
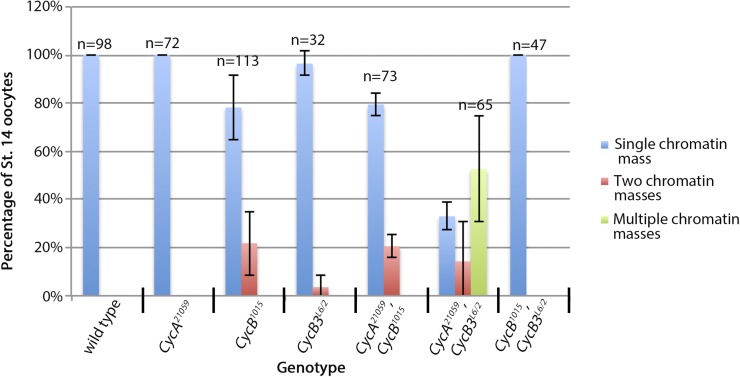
Contribution of mitotic cyclins to metaphase I arrest. Stage 14 oocytes were labeled with Oligreen to detect DNA and with the X-cent FISH probe. The graph shows the percentage of stage 14 oocytes that have a single chromatin mass, two chromatin masses or multiple chromatin masses. Results are from two independent experiments. Error bars indicate SEM. FISH, fluorescence *in situ* hybridization; St., stage; X-cent, probe specific to the pericentric region of the X-chromosome.

After NEB, the first meiotic spindle is assembled and the oocyte arrests in metaphase I until ovulation. To analyze the role of each cyclin-Cdk1 complex in achieving or maintaining this metaphase I arrest, we collected stage 14 oocytes, removed chorion and vitelline membranes to permit immunostaining, then probed these oocytes for Tubulin and DNA, and performed FISH using a probe specific to the pericentric region of the X-chromosome (hereafter referred to as the X-cent FISH probe) (Dernburg 2000) (Figure 3). To obtain larger numbers of oocytes for quantification, we separately examined oocytes in which Tubulin immunostaining was omitted (allowing us to also omit the chorion and vitelline membrane removal step) (Figure 4). In wild-type stage 14 oocytes, the chromatin appeared as a single compact mass at the midzone of an acentriolar spindle. The X-cent FISH probe revealed the two X-chromosome centromeres oriented toward opposite poles (Figure 3A and Figure 4). This biorientation of homologs is due to centromere pairs being pulled toward either pole while at the same time being held together by the combined effects of crossing over and sister chromatid cohesion (McKim *et al.* 2002). *CycB3^L6/2^* oocytes arrested normally in stage 14, with a single compact chromatin mass and two X-cent FISH dots oriented toward the poles (Figure 3B and Figure 4). *CycA^21059^* oocytes also contained a single compact mass of chromatin as in the wild type, suggesting that they also stably arrest in metaphase I (Figure 3, C and D and Figure 4). However, biorientation was disrupted in 45% of *CycA^21059^* oocytes. In most cases (34% of *CycA^21059^* oocytes), both X-cent FISH foci were oriented toward the same pole (Figure 3C and Supplemental Material, Figure S1), while in 11% of *CycA^21059^* oocytes one or both FISH dots was centered within the chromatin mass (Figure 3D and Figure S1). X-chromosome biorientation was unaffected in wild-type, *CycB^1015^*, and in *CycB3^L6/2^* oocytes (Figure S1).

### Cyclin B is required for maintenance of metaphase I arrest

Chromatin and X-cent FISH labeling of *CycB^1015^* oocytes revealed that the majority arrest normally in metaphase I. However, 20% contained two distinct chromatin masses, with a single X-cent FISH dot in each (Figure 3E and Figure 4). This phenotype could be due to precocious separation of homologs, though we cannot rule out the possibility that homologs remain paired through stretched out chromatin strands that we do not detect. A similar split chromatin phenotype was seen in *CycB^1896^* oocytes at 26% (*n* = 145) (Figure 3F), and in *CycB^−/−^*;*hs-CycB* oocytes at 19% (*n* = 128). *CycB^1896^* and *CycB^−/−^*;*hs-CycB* oocytes also occasionally displayed more complex and variable phenotypes including spindle morphology defects, multiple chromatin masses, and random distribution of the two X-chromosomes (Figure 3, G and H and data not shown). Therefore, CycB is at least partially required for maintenance of the metaphase I arrest and may also be required for organization of the meiosis I spindle.

### Double knockdown phenotypes suggest that CycB functions independent of CycA and in opposition to CycB3 in meiosis I

We next examined stage 14 oocytes in which two of the three mitotic cyclins were depleted, starting with the *CycA,B* combination. Of stage 14 *CycA^21059^*,*CycB^1015^* oocytes, 21% appeared to have undergone precocious anaphase, as judged by the presence of two distinct chromatin masses (Figure 3I and Figure 4). This frequency is similar to that of *CycB^1015^* alone (Figure 4). *CycA^21059^*,*CycB^1015^* oocytes also displayed biorientation defects, similar to those seen in *CycA^21059^* (Figure 3J). Therefore, it appears that double knockdown of *CycA* and *CycB* produces a phenotype that is equal to the sum of the two single knockdowns. This implies that the two genes act independently. As discussed earlier, it is possible that we fail to detect synergy between *CycA* and *CycB* knockdowns due to weaker expression of the two *UAS-RNAi* lines when expressed together.

We next examined oocytes knocked down for *CycB* and mutant for *CycB3*. As described above, *CycB* knockdown results in a partially penetrant failure to arrest in metaphase I in stage 14 of oogenesis, while *CycB3* mutants arrest normally at this stage. Interestingly, *CycB^1015^*,*CycB3^L6/2^* oocytes invariably contained a single meiotic spindle with a single chromatin mass, indicating that they arrest normally in metaphase I (Figure 3K and Figure 4). Therefore, the failed metaphase I arrest that occurs upon *CycB* knockdown is completely suppressed by homozygous loss of *CycB3*, arguing that CycB and CycB3 have opposing functions.

### CycA and CycB3 act redundantly to prevent rereplication in meiosis I

We next examined stage 14 oocytes from *CycA,CycB3* double knockdown females. Of the stage 14 *CycA^21059^*,*CycB3^L6/2^* oocytes, 33% contained a single chromatin mass suggesting that they were in metaphase I. However, the chromatin mass often appeared asymmetric, as if not all chromosomes were properly aligned at the spindle midzone (Figure 3L and Figure 4). Consistent with this, FISH often revealed failed biorientation of X-chromosomes (Figure 3L). In 14% of *CycA^21059^*,*CycB3^L6/2^* oocytes, the chromatin mass appeared to have broken up into two distinct masses (Figure 4). These were often different sizes, and the two X-cent FISH dots were randomly distributed between the two chromatin masses (data not shown). The majority of *CycA^21059^*,*CycB3^L6/2^* oocytes (53%) had three or more chromatin masses (Figure 4). Of these oocytes, a minority (17%) appeared similar to the above except for the increased number of distinct chromatin masses (Figure 3M). In 83% of the oocytes that had three or more chromatin masses (46% of all oocytes examined), there were more than four X-cent FISH dots, indicating that the meiotic chromatin had rereplicated (Figure 3N). In these oocytes, the chromatin was associated with highly abnormal spindles or disorganized microtubule arrays (Figure 3N). Therefore, CycA and CycB3 function redundantly to organize chromosomes on the meiosis I spindle and to inhibit DNA replication in meiosis I.

### Roles of mitotic cyclins in the completion of meiosis

**Figure 5:**
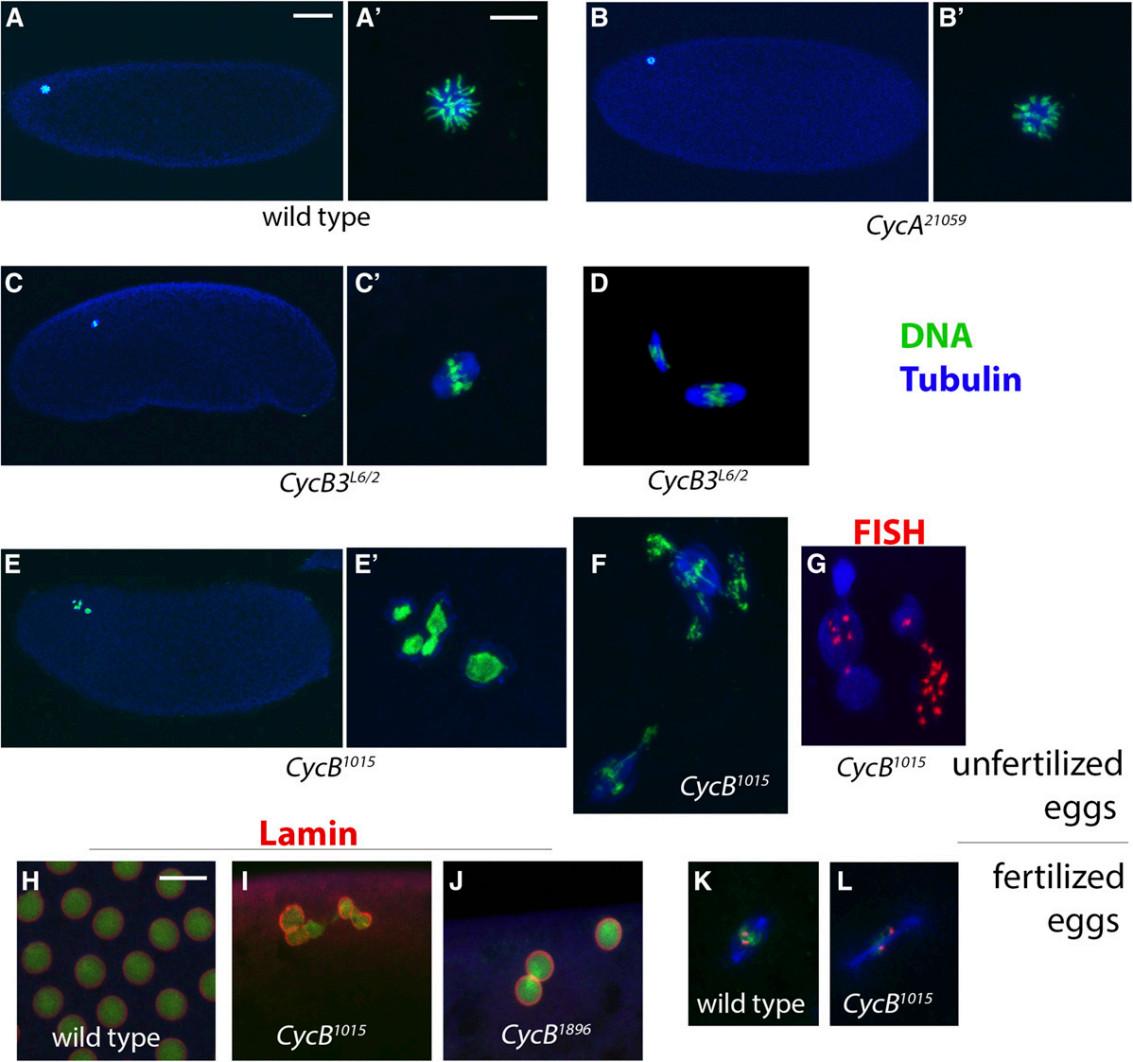
Postovulation requirements for the mitotic cyclins. (A–G) 0–2 hr eggs from unfertilized females, immunostained to detect Tubulin (blue) and chromatin (using α-Histone H3 antibody) in green (except G in which Histone staining is omitted and the X-cent FISH probe (red) is used. Images labeled (A’-C’, E’) are magnified views of the images in (A-C, E), respectively. (A and B) Polar bodies from wild-type and *CycA^21059^* eggs. (C and D) *CycB3^L6/2^* eggs in meiosis I (C) and meiosis II (D). (E) *CycB^1015^* egg in postmeiotic interphase. (F) *CycB^1015^* egg in meiosis II with highly aberrant spindles. (G) *CycB^1015^* egg with an unknown meiotic phenotype. X-cent FISH reveals multiple foci, indicative of rereplication. (H–J) Fertilized 0–2 hr eggs labeled for Lamin (red), Tubulin (blue), and DNA (green). (H) Wild-type syncytial stage embryo in interphase. (I and J) *CycB^1015^* and *CycB^1896^* embryos in postmeiotic interphase. (K and L) Internal mitotic spindle from fertilized wild-type and *CycB^1015^* eggs labeled for Tubulin (blue), Histone H3 (green), and X-cent FISH (red). The FISH probe detects two distinct foci. Scale bar in (A) = 50 μm and applies to (A), (B), (C), and (E). Scale bar in (A’) and (H) = 10 μm and applies to (A’), (B’), (C’), (D), and (E’–L). FISH, fluorescence *in situ* hybridization; X-cent, probe specific to the pericentric region of the X-chromosome.

**Figure 6:**
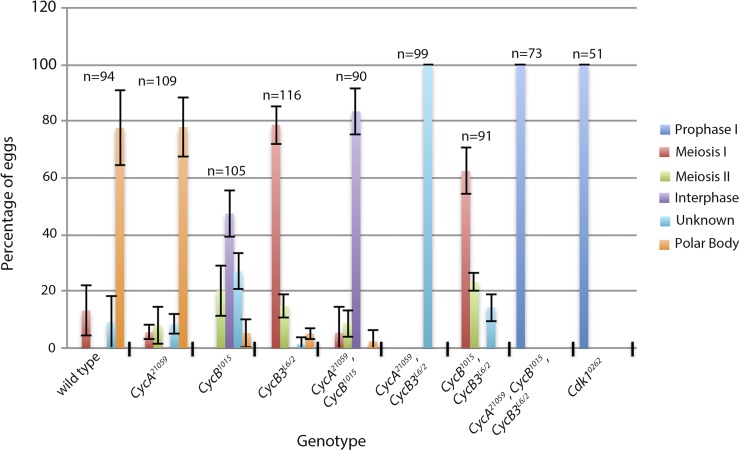
Postovulation phenotypes in cyclin and *Cdk1* knockdown eggs. Unfertilized eggs from each genotype were collected over a 2 hr period and probed for Tubulin and Histone H3 to label chromatin. Graph shows the percentage of eggs that appear to be in prophase I, meiosis I, meiosis II, postmeiotic interphase, unknown phenotype, or with polar bodies. Approximately 50% of *CycB^1015^*,*CycB3^L6/2^* eggs contain one or two nuclei, but these were classified as meiosis I or II-arrested, respectively (see text). The graph represents data from three independent experiments (two in the case of *Cdk1^0262^*). Error bars indicate SEM. Cdk, cyclin-dependent kinase; Cyc, cyclin.

To determine how loss of the individual mitotic cyclins affects the completion of meiosis following ovulation, we first examined 0–2 hr unfertilized eggs probed for Tubulin and Histone H3 to detect chromatin. Representative phenotypes are shown in Figure 5 and quantification is presented in Figure 6. Eggs from *CycA* knockdown females appeared to complete meiosis. Of the unfertilized eggs from *CycA^21059^*, 78% contained a polar body, the same percentage as in the wild type (Figure 5, A and B and Figure 6). Though meiosis was able to proceed to completion, FISH using the X-cent probe suggested that it did not proceed normally. In fertilized wild-type eggs, the polar body contained three X-cent FISH signals corresponding to the three polar body nuclei. The other meiotic product joins the male pronucleus to form the zygote. In fertilized *CycA^21059^* eggs, many polar bodies had two or four X-cent FISH signals (Figure S2). This apparent variability in the number of X-chromosomes in polar bodies could be due to defects in homolog segregation in meiosis I, as would be expected from the biorientation defects seen in stage 14 oocytes (Figure 3 and Figure S1).

**Figure 7:**
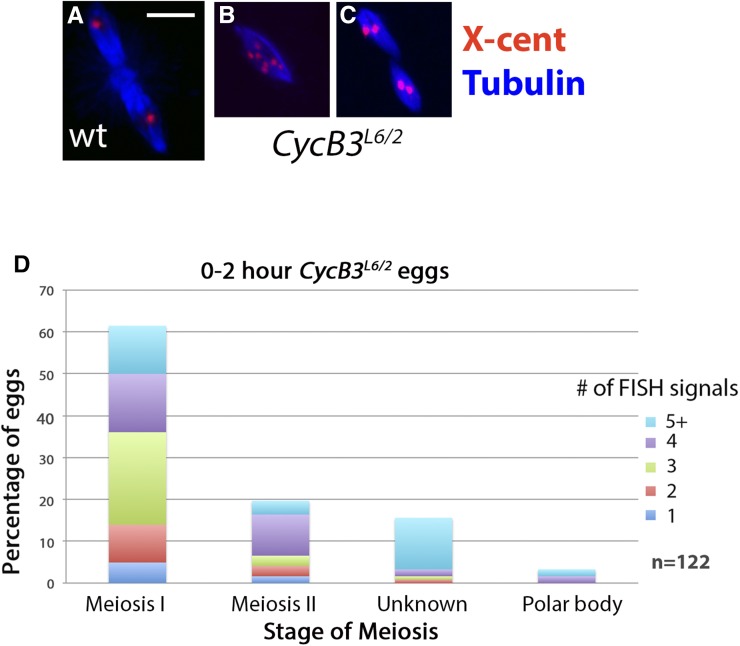
*CycB3* mutants arrest with separated sister chromatids. (A–C) Meiotic figures from wild-type and *CycB3^L6/2^* eggs, labeled for Tubulin (blue) and X-cent FISH (red). The wild-type egg (A) has two spindles with a single X-cent FISH dot per spindle, and thus is in metaphase II. The meiosis I-arrested *CycB3^L6/2^* egg (B) has seven distinct X-cent FISH dots, indicative of sister chromatid separation and rereplication. The meiosis II *CycB3^L6/2^* egg (C) has two X-cent FISH dots per spindle and thus is in anaphase II. (D) Quantification of X-cent FISH dots per meiotic figure in 0–2 hr *CycB3^L6/2^* eggs. Data are pooled from three independent experiments. Scale bar in (A) = 10 μm. Cdk, cyclin-dependent kinase; Cyc, cyclin; FISH, fluorescence *in situ* hybridization; X-cent, probe specific to the pericentric region of the X-chromosome.

**Figure 8:**
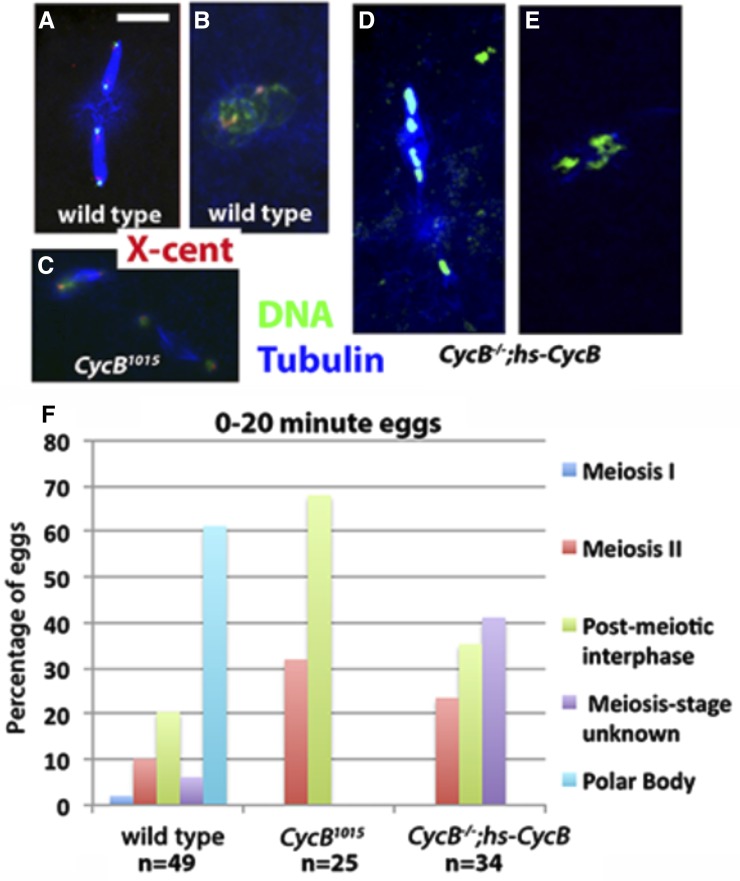
CycB is required for normal progression through meiosis II and for polar body formation. (A–E) Fertilized eggs collected over a 20 min period from wild-type and *CycB* knockdown females as indicated, probed for Tubulin (blue) and Histone H3 to label chromatin (green). X-cent FISH (red) is included in (A–C). (A) Wild-type egg in anaphase II. (B) Wild-type egg in postmeiotic interphase. Nuclei are clustered together in preparation for polar body formation. (C) *CycB^1015^* egg in meiosis II. One of the meiotic spindles appears to be in anaphase while the other is in metaphase. Spindle morphology is also disrupted. (D) *CycB^−/−^;hs-CycB* egg in meiosis II. The two spindles have different amounts of associated chromatin, suggesting that anaphase I was unequal. (E) *CycB^−/−^;hs-CycB* egg that appears to be in postmeiotic interphase. (F) Graph showing percentage of 0–20 min eggs in the different meiotic stages. The graph represents results from multiple egg collections that were pooled together in a single experiment. Cdk, cyclin-dependent kinase; Cyc, cyclin; FISH, fluorescence *in situ* hybridization; X-cent, probe specific to the pericentric region of the X-chromosome.

0–2 hr eggs from *CycB3^L6/2^* females typically contain either one or two spindles in the dorsal anterior of the egg, indicative of an arrest in meiosis I or meiosis II (Jacobs *et al.* 1998; Yuan and O’Farrell 2015) (Figure 5, C and D and Figure 6). The relative frequency of eggs with one *vs.* two spindles does not appear to differ when we compared 0–20, 20–40, and 40–60 min egg-lays (Figure S3), arguing that the eggs that are in meiosis I and meiosis II are stably arrested at that stage as opposed to progressing slowly through meiosis. To further characterize the *CycB3^L6/2^* meiotic arrest, we examined eggs labeled with the X-cent FISH probe. Wild-type eggs in metaphase II have two X-cent FISH foci, one per spindle (Figure 7A). These resolve into two foci per spindle (four in total) upon sister chromatid segregation in anaphase II (see Figure 8A). In *CycB3^L6/2^*, 59% of meiosis I-arrested oocytes and 63% of meiosis II-arrested oocytes have either three or four distinct FISH dots in the spindle, indicating that sister centromeres separate from each other in most oocytes, even those that appear to be in meiosis I (Figure 7, C and D). These findings suggest that CycB3 is necessary for spindle behavior and chromosome segregation in anaphase, but not for the release of sister chromatid cohesion. Alternatively, it may be that CycB3 is indeed required for the timely release of sister chromatids but that cohesion fatigue (Daum *et al.* 2011) leads to their eventual separation in *CycB3* mutants.

Approximately 19% of meiosis I and 17% of meiosis II eggs from *CycB3^L6/2^* had more than four X-cent FISH dots, indicative of rereplication (Figure 7, B and D). Further, 16% of *CycB3^L6/2^* eggs displayed variable and difficult to characterize phenotypes such as presence of more than two spindles or microtubule arrays with associated chromatin (Figure 7D). The rereplication and uncharacterizable phenotypes tended to occur together (Figure 7D), and may both be secondary effects of prolonged meiotic arrest.

### Cyclin B is required for polar body formation

We next examined eggs from *CycB* knockdown females. As described above, 78% of wild-type eggs collected over a 2 hr period contain polar bodies. In contrast, only 6% of *CycB^1015^* eggs collected over the same time period had recognizable polar bodies (Figure 6). 20% had two spindles with abnormal morphology, suggesting a meiosis II delay or arrest (Figure 5F and Figure 6), while 47% appeared to be in postmeiotic interphase (Figure 5E and Figure 6). Often the nuclei were clustered together, as often seen in wild-type eggs just prior to polar body formation. The number of nuclei varied, possibly due to segregation defects in meiosis. Nuclei often appeared to be surrounded by microtubules (Figure 5E), possibly as a result of prolonged arrest in interphase. The remaining 27% of *CycB^1015^* eggs had more complex phenotypes and therefore could not be categorized with respect to meiotic stage. These eggs contained variable sized spindles or microtubule arrays, often associated with large amounts of chromatin. FISH using the X-cent FISH probe revealed multiple distinct foci in most of these eggs, indicating that rereplication had occurred (Figure 5G).

To confirm that the eggs we had categorized as being in postmeiotic interphase indeed contained nuclei, we probed 0–2 hr fertilized eggs with antibodies against Lamin (Figure 5, H–J). In the wild type, Lamin was never detected in association with the polar body (data not shown), though it could be readily detected on mitotic nuclei in interphase (Figure 5H). Of the *CycB^1015^* eggs, 38% (10/26) had Lamin staining associated with the female meiotic products (Figure 5I). The other *CycB* RNAi line, *CycB^1896^*, also appeared to arrest in postmeiotic interphase with Lamin-positive nuclei (Figure 5J).

Interestingly, while the polar body nuclei in *CycB^1015^* eggs did not undergo NEB, if the eggs were fertilized they usually contained one or more mitotic spindles in the egg interior. From the above-described collections of fertilized *CycB^1015^* eggs probed for Lamin, Tubulin, and DNA, 92% (24/26) had identifiable (though often aberrant) mitotic spindles in the egg interior, and no associated Lamin signal. Mitotic spindles were not found in unfertilized *CycB^1015^* eggs, and therefore the spindles and associated chromatin are presumably derived from the male pronucleus. We used the X-cent FISH probe on *CycB^1015^* embryos to determine whether or not a female pronucleus also contributes to these mitotic spindles. If it does, the mitotic spindle or spindles in all embryos would have at least one X-chromosome. If the female does not contribute, half of all embryos will have no X-chromosome in their mitotic spindles (in cases where the embryo is YO). In the wild type, 95% (± 7.9, *n* = 40) of eggs had at least one X-cent FISH signal in their mitotic spindle or spindles (Figure 5K). A similar percentage was seen in *CycB^1015^* embryos (93% ± 4.9, *n* = 42) (Figure 5L). Therefore, in *CycB^1015^*, the male and female pronuclei appear to be able to come together, undergo NEB, and form the zygote.

As described above, 27% of *CycB^1015^* eggs from 2 hr egg lays contained apparently disorganized arrays of chromatin and microtubules (Figure 6). Virtually all 0–2 hr eggs from *CycB^−/−^*;*hs-CycB* females had similar complex phenotypes (data not shown, but see below). It is possible that these phenotypes result from instability of either the meiosis II or postmeiotic interphase arrests. If so, they should be less frequent in eggs from shorter egg lays. To test this, we examined eggs from 20 min egg collections (Figure 8). Most wild-type 0–20 min eggs had completed meiosis and contained polar bodies, though a small percentage were still in meiosis or in postmeiotic interphase (Figure 8, A, B, and F). In 20 min collections from *CycB^1015^*, we never observed eggs with polar bodies. Further, we no longer observed complex phenotypes such as those seen in the 2 hr egg collections. Instead, all eggs were either in meiosis II or postmeiotic interphase (Figure 8, C and F). Meiosis II spindle morphology and chromatin distribution on the spindles appeared abnormal (Figure 8C), suggesting that meiotic spindle defects in *CycB^1015^* eggs are not simply a result of prolonged arrest.

We also examined 0–20 min eggs from *CycB^−/−^*;*hs-CycB*. Of these eggs, 53% had complex and uncharacterizable phenotypes (compared to 100% in 2 hr egg lays) (Figure 8F). All other eggs appeared to be either in meiosis II or in postmeiotic interphase (Figure 8, D–F). All meiosis II eggs from *CycB^−/−^*;*hs-CycB* had highly aberrant spindle morphology and the chromatin distribution on these spindles appeared unequal (Figure 8D). Overall, it appears that the *CycB^−/−^*;*hs-CycB* phenotype is similar to but stronger than *CycB^1015^*. Both alleles produce a delay or arrest in meiosis II with aberrant spindle morphology, and an arrest prior to polar body formation. These arrests are not stable and the meiotic products rereplicate and become increasingly disorganized. Therefore, CycB is at least partially required for meiotic spindle organization and completion of the second meiotic division, and is essential for polar body formation.

### Redundancies among mitotic cyclins in the completion of meiosis

**Figure 9:**
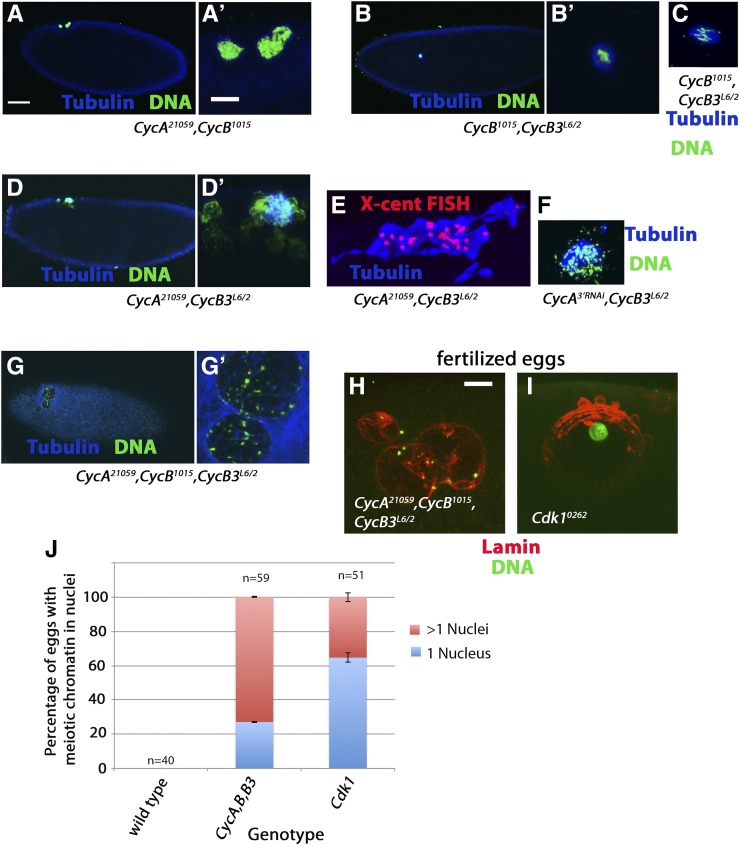
Redundant roles for mitotic cyclins in the completion of meiosis. (A–G’) Unfertilized 0–2 hr eggs labeled for Tubulin (blue) and Histone H3 to label chromatin (green), except (E) which is labeled for Tubulin (blue) and X-cent (red). Images (A’, B’, D’ and G’) are zoomed-in views of (A, B, D and G), respectively. (A) *CycA^21059^*, *CycB^1015^* egg in postmeiotic interphase. (B) *CycB^1015^*,*CycB3^L6/2^* egg arrested with a single nucleus. (C) *CycB^1015^*,*CycB3^L6/2^* egg arrested with a single meiosis I spindle. (D and D’) *CycA^21059^*,*CycB3^L6/2^* egg with large masses of chromatin and associated microtubules. (F) *CycA^3′RNAi^*,*CycB3^L6/2^* egg with scattered chromatin and associated microtubule arrays. (G) *CycA^21059^*, *CycB^1015^*,*CycB3^L6/2^* egg with two large nuclei. (H and I) Unfertilized 0–2 hr eggs labeled for Lamin (red) and Histone H3 (green). (H) *CycA*,*B*,*B3* triple knockdown oocyte with three large nuclei. (I) *Cdk1^0262^* egg with a single large nucleus. Scale bar in (A) = 50 μm and applies to (A), (B), (D), and (G). Scale bars in (A’) and (H) = 10 μm and apply to all other panels. (J) Graph showing percentage of wild-type, *CycA^21059^*, *CycB^1015^*,*CycB3^L6/2^*, and *Cdk1^0262^* eggs with Lamin-positive nuclei (0% in wild-type, 100% in both knockdowns), and percentage that have one nucleus *vs.* two or more nuclei. Results for *CycA^21059^*, *CycB^1015^*,*CycB3^L6/2^*, and *Cdk1^0262^* are based on two independent experiments, and error bars indicate SEM. Results for wild-type come from a single experiment. Cdk, cyclin-dependent kinase; Cyc, cyclin; FISH, fluorescence *in situ* hybridization; X-cent, probe specific to the pericentric region of the X-chromosome.

We next examined embryos from double knockdown females. Over 80% of *CycA^21059^*,*CycB^1015^* eggs from 2 hr collections appeared to be in postmeiotic interphase (Figure 6 and Figure 9A). This phenotype was also seen in *CycB^1015^* single knockdown eggs. However, unlike *CycB^1015^* alone, *CycA^21059^*,*CycB^1015^* did not produce a delay or arrest in meiosis II (Figure 6). It is not clear if this reflects a specific effect of *CycA* knockdown or if it is merely due to lower *CycB* knockdown efficiency in the presence of a second *UAS-RNAi* line. Overall, considering both pre- and postovulation phenotypes, the double knockdown appears more-or-less equivalent to the sum of the two single knockdowns. While the incomplete nature of RNAi knockdowns makes it difficult to conclude with certainty, our findings suggest that CycA-Cdk1 and CycB-Cdk1 have largely nonoverlapping functions in meiosis.

We next examined eggs in which both *CycB* and *CycB3* are knocked down. Approximately 50% of the eggs from *CycB^1015^*,*CycB3^L6/2^* arrested in either meiosis I or meiosis II, similar to *CycB3* single mutants (Figure 6 and Figure 9C). The remaining 50% of eggs contained either one or two masses of condensed chromatin that appeared to be within nuclear envelopes and surrounded by microtubules (Figure 9B). The position of these nuclei at the dorsal anterior of the egg and their number (either one or two) suggest that they are derived from meiosis I or II figures respectively. We speculate that *CycB^1015^*,*CycB3^L6/2^* eggs arrest in meiosis I or II (like *CycB3^L6/2^* alone), and that low Cdk1 activity results in nuclear envelopes reassembling around the meiotic chromatin. If we count these eggs as having arrested in meiosis I and meiosis II, respectively, the frequency of meiosis I arrest is 62% and meiosis II is 23%. This is similar to that of *CycB3^L6/2^* alone (Figure 6). Therefore, it appears that the *CycB,B3* double knockdown shows the same meiosis I or meiosis II arrest as seen in *CycB3* mutants alone. This suggests that CycB and CycB3 are largely nonredundant in meiosis.

We next examined eggs from females depleted of both CycA and CycB3. All eggs from *CycA^21059^*,*CycB3^L6/2^* females contained large masses of chromatin and associated microtubules (Figure 9D). FISH revealed multiple X-chromosome foci within these chromatin masses (Figure 9E). Overall, the phenotype of *CycA^21059^*,*CycB3^L6/2^* eggs appears similar to but stronger and more penetrant than that seen in mature oocytes. Importantly, we also saw rereplication and disorganized microtubule arrays (though less pronounced) in eggs in which *CycB3^L6/2^* is combined with the weaker *CycA* RNAi line, *CycA^3′RNAi^* (Figure 9F). The fact that two independent *CycA* RNAi lines produce the same meiosis defects in combination with *CycB3* mutants supports the conclusion that the two lines specifically affect the *CycA* gene.

Finally, we examined eggs from females in which all three mitotic cyclins have been knocked down. As described earlier, *CycA^21059^*,*CycB^1015^*,*CycB3^L6/2^* oocytes are delayed in NEB, but by stage 14 only 14% still have a detectable nucleus (Figure 2). However, 100% of unfertilized eggs from *CycA^21059^*,*CycB^1015^*,*CycB3^L6/2^* females contained one or more large nuclei in the dorsal anterior of the egg, a site that corresponds to the location of the original prophase I nucleus (Figure 9, G and J). Lamin staining confirmed the presence of nuclear envelopes (Figure 9H). Of these eggs, 27% contained a single large nucleus while the remaining eggs had >1 (typically 2–4) nuclei of often differing sizes (Figure 9, G and J). Therefore, it seems that in *CycA^21059^*,*CycB^1015^*,*CycB3^L6/2^*, NEB occurs in late oogenesis, but nuclear envelopes reassemble shortly after ovulation. We speculate that eggs with multiple nuclei are those in which NEB occurred prior to ovulation. Chromatin then dispersed, and each chromatin mass become surrounded by its own nuclear envelope after ovulation.

*Cdk1^0262^* eggs also invariably contained one or more large dorsal anterior nuclei (Figure 9, I and J), suggesting that NEB (if it occurs) is followed by the reassembly of nuclear envelopes after ovulation. While 73% of *CycA^21059^*,*CycB^1015^*,*CycB3^L6/2^* eggs had more than one nucleus, only 35% of *Cdk1^0262^* eggs had more than one (Figure 9, I and J). This is consistent with the idea that multiple nuclei are the result of NEB having occurred prior to ovulation, followed by dispersal of chromatin and eventual reassembly of nuclear envelopes around the dispersed chromatin. *Cdk1* knockdown results in relatively fewer oocytes undergoing NEB and thus fewer oocytes in which chromatin dispersal occurs.

An alternative explanation for the appearance of nuclei in *CycA*,*B*,*B3* triple knockdown and *Cdk1* knockdown eggs is that meiosis proceeds to completion and the meiotic products arrest in the postmeiotic interphase that precedes polar body formation, similar to what appears to happen in the *CycB* single knockdown. We cannot yet discount this possibility, but the following observations argue against it. First, we never saw meiotic spindles in *CycA^21059^*,*CycB^1015^*,*CycB3^L6/2^* or *Cdk1^0262^* eggs (Figure 6), suggesting that they never progress beyond NEB. Second, the *CycB3* single and double knockdown combinations produced an arrest prior to the completion of meiosis. It seems unlikely that triple knockdown eggs would be able to complete meiosis if these single and double knockdowns do not. We conclude that knockdown of *Cdk1* or all three of its cyclin partners results in a delay or complete failure of NEB. Those oocytes that undergo NEB prior to ovulation end up reassembling nuclear envelopes shortly after ovulation.

## Discussion

Using a combination of RNAi knockdown and mutants, we have analyzed the genetic requirements for the three mitotic cyclins in *Drosophila* meiosis. Our conclusions about cyclin requirements are, in part, based on incomplete knockdown, and it is possible that stronger or novel phenotypes will be revealed with stronger gene knockdowns. Nonetheless, we are able to identify important unique and shared functions for each cyclin-Cdk1 complex.

### CycB has a possible role in homolog cohesion

The two different *CycB* RNAi lines, as well as the conditional allele *CycB^−/−^*;*hs-CycB*, produce a partially penetrant phenotype in which the metaphase I arrest in stage 14 of oogenesis is not maintained. The most common observation is a splitting of the chromatin into two distinct and generally equivalent masses. This could reflect a role for CycB in maintaining cohesion between homologs. Homolog cohesion depends on the combined effect of crossovers between homologs and sister chromatid cohesion mediated by Cohesin complexes (reviewed in Revenkova and Jessberger 2005). Anaphase I is initiated when Cohesins distal to crossover sites are disassembled. This depends on the activation of Separase, a protease that cleaves one of the Cohesin subunits. Separase activity is inhibited prior to anaphase I by inhibitory binding of Securin (Pim in *Drosophila*), a protein that is targeted for destruction by the APC/C at anaphase I onset. In *Xenopus* oocyte extracts, CycB1-Cdk1 also contributes to Separase inhibition, both by phosphorylation of Separase and by inhibitory binding (Gorr *et al.* 2005). The roles of Separase and Securin in meiosis appear to be conserved in *Drosophila* (Guo *et al.* 2016), though the Separase target within the Cohesin complex has yet to be identified (Urban *et al.* 2014; Guo *et al.* 2016). Our results suggest that the CycB-Cdk1 role in Separase inhibition may also be conserved. Of note in this respect, the split chromatin phenotype occurs in only 20% of *CycB^1015^* oocytes (and at 19% in the strong hypomorph *CycB^−/−^*;*hs-CycB*), and the penetrance of this phenotype does not go up when *CycA* or *CycB3* are also depleted. While low penetrance may relate to incomplete knockdown, another explanation is that CycB-Cdk1 functions redundantly with Securin to keep Separase inactive and thus prevent precocious anaphase.

### CycB-Cdk1 prevents anaphase while CycB3-Cdk1 promotes anaphase in meiosis

The split chromatin phenotype in *CycB* knockdown oocytes is completely suppressed by homozygous loss of *CycB3*. *CycB3* mutant eggs arrest in anaphase I (or anaphase II) following ovulation (Jacobs *et al.* 1998) (Figure 5), suggesting a simple interpretation of this genetic suppression: that CycB3 is required not only for the physiological anaphase that occurs upon ovulation, but also for the precocious anaphase that occurs upon *CycB* knockdown. CycB-Cdk1 and CycB3-Cdk1 thus appear to have somewhat opposing functions in meiosis: CycB prevents anaphase and CycB3 promotes anaphase. This may be similar to the situation in the syncytial embryo where CycB3 promotes anaphase, possibly by stimulating the APC/C-mediated destruction of the other two mitotic cyclins (Yuan and O’Farrell 2015). CycB3 is also required for anaphase progression in *Caenorhabditis elegans* and in mouse oocytes (van der Voet *et al.* 2009; Zhang *et al.* 2015), suggesting a conserved function in APC/C activation.

### CycA promotes biorientation in meiosis

CycA, the only mitotic cyclin that is essential for viability in *Drosophila*, does not appear to be essential for the completion of meiosis. However, it plays a critical role in biorientation of homologs in meiosis I. Biorientation in *Drosophila* female meiosis appears to occur in two steps (Radford *et al.* 2015). First, chromosomes make side-on attachments to the overlapping microtubules of the central spindle. The CENP-E microtubule motor associates with the central spindle and with kinetochores to bring the chromosomes toward opposite poles. In the second step, microtubules make head-on attachments to kinetochores (Radford *et al.* 2015). The biorientation defects seen in *CycA^21059^* resemble those seen with weak alleles of *Incenp* and *Aurora B*, central spindle components that are implicated in the first step (Radford *et al.* 2012). Cdk1 appears to phosphorylate Incenp (Goto *et al.* 2006), suggesting one possible mechanism by which CycA-Cdk1 could be involved in biorientation. Indirect evidence also suggests a role for CycA in the second step in biorientation. CycA-Cdk1 has been shown to destabilize incorrect head-on attachments between kinetochores and microtubules in mitosis (Kabeche and Compton 2013). It is possible that it has a similar role in *Drosophila* meiosis.

While *CycA* knockdown disrupts homolog biorientation, the chromosomes remain within a single compact mass. When CycA and CycB3 are both depleted, the chromatin often appears less compact, or separates into two or more chromatin masses. The scattered or dispersed chromatin, often accompanied by spindle abnormalities, is reminiscent of phenotypes seen in *twine*, *Endos*, and *Gwl* mutants (Archambault *et al.* 2007; Von Stetina *et al.* 2008), genes that are implicated in Cdk1-mediated phosphorylation. *Twine* encodes one of two *Drosophila* Cdc25 homologs that directly activate Cdk1 via dephosphorylation. Endos and Gwl function in a pathway to inhibit PP2A, a phosphatase that dephosphorylates Cdk1 substrates (Rangone *et al.* 2011; Wang *et al.* 2011). Therefore, Twine, Gwl, and Endos all collaborate to support the activity of CycA-Cdk1 and CycB3-Cdk1 complexes in meiosis. We note that *CycA*,*CycB3* double knockdown oocytes also display a rereplication phenotype that does not occur in *Gwl* mutants (Archambault *et al.* 2007), and that has not been reported for *twine* or *Endos* mutants. This may simply reflect a greater effect on Cdk1 activity in the *CycA*,*CycB3* double knockdown compared to *Gwl*, *twine*, or *Endos* mutants.

### Cyclin-Cdk1 control of NEB in meiosis and in polar body formation

We found that cyclins A, B, and B3 all function redundantly to promote NEB in *Drosophila* oocytes, with CycA having the most important role and CycB playing a relatively minor role. This was surprising because, in vertebrates, Cyclin B (mainly CycB1) is the essential Cdk partner for meiotic maturation (Labbe *et al.* 1989; Gui and Homer 2013). CycA (CycA2 specifically) plays a relatively minor role (Touati *et al.* 2012) and CycB3 has no known role in meiotic maturation. It is possible that our failure to detect a nonredundant role for CycB in *Drosophila* oocyte maturation is simply due to incomplete knockdown, though in the case of *CycB^−/−^;hs-CycB*, 97% of CycB is depleted and NEB timing is still not affected.

It is interesting that, in *Cdk1^0262^* and *CycA*,*B*,*B3* triple knockdowns, NEB often occurs prior to ovulation but the nuclear envelope inevitably reassembles after ovulation. One of the key events triggered by ovulation is the activation of the meiosis-specific APC/C-Cort ubiquitin ligase that targets mitotic cyclins for destruction (Swan and Schupbach 2007; Pesin and Orr-Weaver 2007). In the background of already reduced Cdk activity due to cyclin or *Cdk1* knockdown, the further drop in Cdk1 activity resulting from APC/C-Cort activation at ovulation may be enough to lead to the reassembly of nuclear envelopes.

While CycA appears to be most important for breakdown of the prophase I nucleus at meiotic maturation, CycB appears to be the most important contributor to the disassembly of polar body nuclei at the completion of meiosis. *CycB* single knockdown as well as *CycA*,*B* double knockdown results in an arrest in the postmeiotic interphase, indicating that CycB-Cdk1 is essential for NEB in these nuclei. Interestingly, about half of the eggs from *CycB*,*B3* double knockdown females arrest with either one or two nuclei. One interpretation of this finding is that loss of CycB leads to nuclear envelope assembly around chromatin that, due to loss of *CycB3*, is arrested in meiosis I or meiosis II. Therefore it appears that, after ovulation, CycB-Cdk1 activity is required to promote or maintain a mitotic-like state, and nuclear envelopes form around chromatin in its absence.

It is not clear why CycB would have a major role in NEB in the egg but not in oocyte maturation, but a simple explanation is that CycB levels are much lower in stage 13 when oocyte maturation occurs. *CycB* mRNA is translationally repressed throughout oogenesis until stage 14 (Benoit *et al.* 2005; Vardy and Orr-Weaver 2007). The increase in relative levels of CycB in mature oocytes and in eggs (Benoit *et al.* 2005; Vardy and Orr-Weaver 2007) may contribute to its renewed ability to promote NEB. We also noted that in the hypomorph for *CycB* (*CycB^1015^*), NEB still occurred in the male and female pronuclei, while not occurring in the polar body nuclei. This may imply a further, spatial control of CycB-Cdk1 activity in the egg.

It is interesting to compare the *CycB* knockdown phenotype to that of *png* (and its partners *gnu* and *plu*). These genes function together to promote *CycB* translation in the embryo (Fenger *et al.* 2000; Lee *et al.* 2001; Vardy and Orr-Weaver 2007). Like *CycB* knockdown, *png* mutants complete meiosis but the meiotic products fail to undergo NEB and form polar bodies (Shamanski and Orr-Weaver 1991). The failure to undergo NEB in *png* mutant polar bodies can be suppressed by overexpression of *CycB* (Lee *et al.* 2001), consistent with the idea that it is the reduction of CycB levels that leads to this effect. In *png* mutants, the meiotic products then undergo dramatic rereplication, something also seen, albeit to a lesser degree, with *CycB* knockdown (Shamanski and Orr-Weaver 1991). Png appears to control the levels of many other maternal proteins, including CycA (Fenger *et al.* 2000; Tadros *et al.* 2007), and it is likely that some of these contribute to the extreme rereplication seen in *png* mutants.

### CycA and CycB3 prevent rereplication in meiosis

Rereplication accompanies the meiotic arrest to different degrees in all three single cyclin knockdowns, but it is most dramatic in the *CycA*,*CycB3* double knockdown, and only this double knockdown leads to rereplication prior to ovulation. A defining feature of meiosis is that the two meiotic divisions occur without an intervening S-phase. This is achieved in large part by the maintenance of a critical threshold of Cdk1 activity throughout the meiotic divisions, thereby inhibiting prereplication complex assembly and preventing DNA replication (Arias and Walter 2007; Irniger 2006). Our results suggest that CycA and CycB3-Cdk1 complexes together maintain the critical threshold of replication-inhibiting Cdk activity during *Drosophila* meiosis.

The distinct requirements for each cyclin in meiosis presumably reflect differences in cyclin concentration over time and space, as well as differences in substrate preference. A major goal of future studies will be to identify individual Cdk1 substrates that correspond to the cyclin loss-of-function phenotypes. It should then be possible to determine the basis for cyclin specificity toward these substrates.

## Supplementary Material

Supplemental Material
