# Supplementary material for: Distinct and Overlapping Requirements for Cyclins A, B, and B3 in Drosophila Female Meiosis
Source: G3 (Bethesda). 2016 Sep 20;6(11):3711–24. doi: 10.1534/g3.116.033050 (PMC5100870; doi:10.1534/g3.116.033050)
Supplement: Supplemental Material [file supp_6_11_3711__index.html]

Distinct and Overlapping Requirements for Cyclins A, B, and B3 in Drosophila Female Meiosis — Supplemental Material 

# Distinct and Overlapping Requirements for Cyclins A, B, and B3 in *Drosophila* Female Meiosis

## Supplemental Material for Bourouh, *et al.*, 2016

**Files in this Data Supplement:**

- Figure S1 - Orientation of X-chromosome centromeres in metaphase I oocytes. (.pdf, 179 KB)
- Figure S2 - *CycA* knockdown leads to variation in number of chromosomes in the polar body. (.pdf, 162 KB)
- Figure S3 - Meiosis phenotypes in timed egg collections from *CycB3L6/2*. (.pdf, 162 KB)
