# Supplementary material for: Distinct and Overlapping Requirements for Cyclins A, B, and B3 in Drosophila Female Meiosis
Source: G3 (Bethesda). 2016 Sep 20;6(11):3711–24. doi: 10.1534/g3.116.033050 (PMC5100870; doi:10.1534/g3.116.033050)
Supplement: Supplemental Material [file supp_g3.116.033050_FigureS1.pdf]

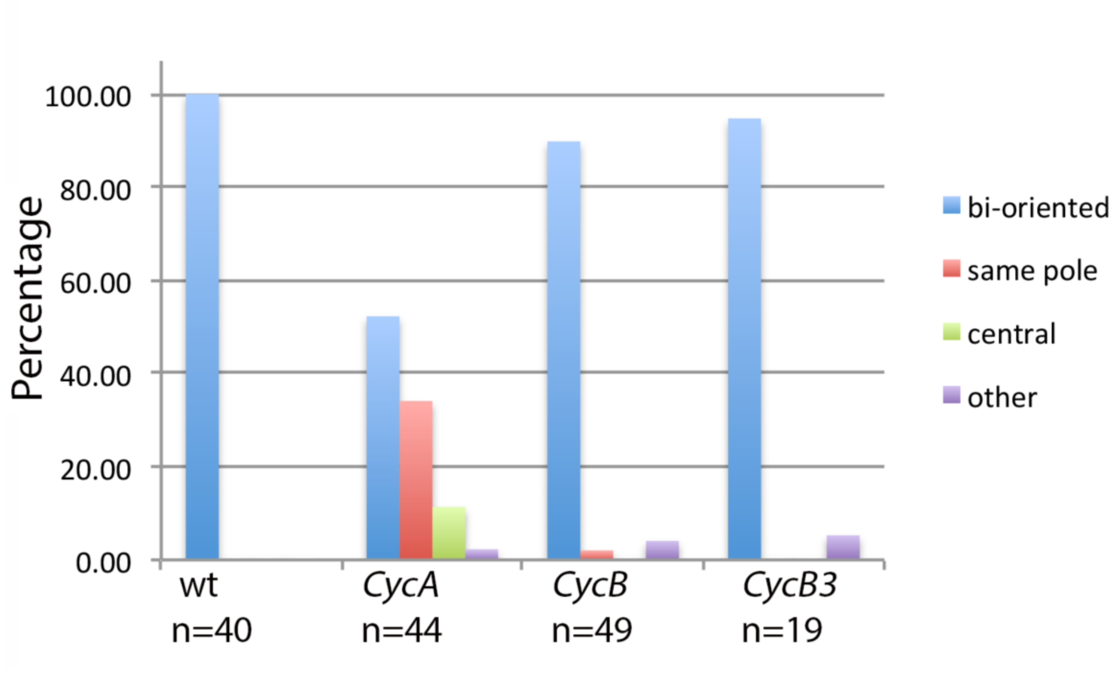

Figure S1.

Orientation of X-chromosome centromeres in metaphase I oocytes. Stage 14 oocytes from wild type, *CycA*^{59}, *CycB*^{1015} and *CycB*$^{L6/2}$ were probed for Tubulin, DNA and peri-centric region of the X-chromosome (X-cent FISH probe). Ooctyes were scored for bi-orientation (FISH signals oriented towards opposing poles), same pole (both FISH dots at the same pole, sometimes appearing as a single dot), central (one or both FISH signals near center of the chromatin mass), or other (meiotic spindle appears abnormal). Results are combined from two independent experiments.
