# Supplementary material for: Distinct and Overlapping Requirements for Cyclins A, B, and B3 in Drosophila Female Meiosis
Source: G3 (Bethesda). 2016 Sep 20;6(11):3711–24. doi: 10.1534/g3.116.033050 (PMC5100870; doi:10.1534/g3.116.033050)
Supplement: Supplemental Material [file supp_g3.116.033050_FigureS2.pdf]

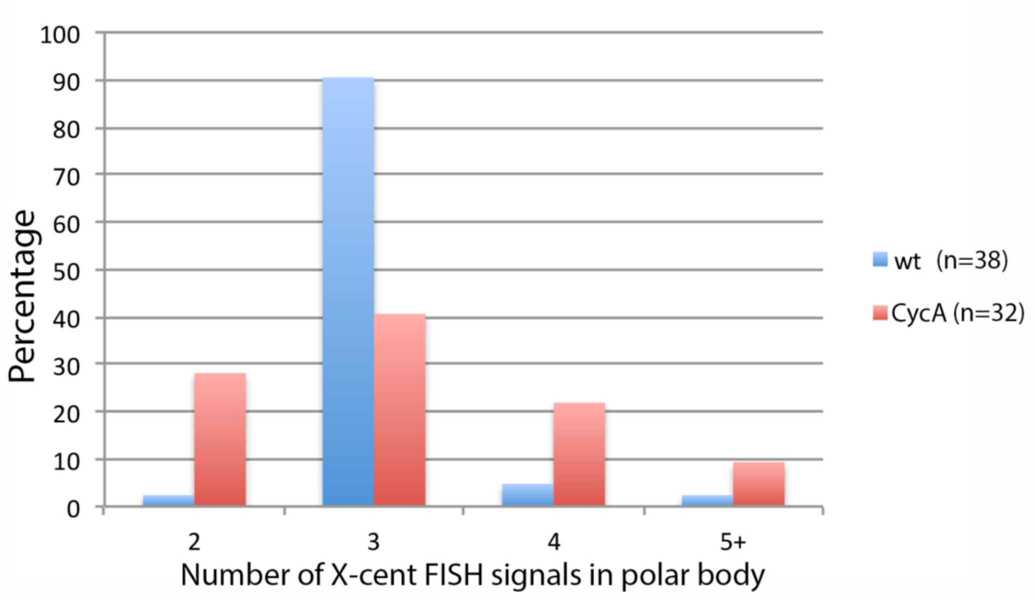

Figure S2

CycA knockdown leads to variation in number of chromosomes in the polar body. 0-2 hour embryos from wild type and from *CycA*^{21059} were probed for DNA, Tubulin and X-chromosome (X-cent FISH probe) and the number of FISH signals in each polar body was determined. Data comes from a single experiment, n values as indicated.
