# Supplementary material for: Distinct and Overlapping Requirements for Cyclins A, B, and B3 in Drosophila Female Meiosis
Source: G3 (Bethesda). 2016 Sep 20;6(11):3711–24. doi: 10.1534/g3.116.033050 (PMC5100870; doi:10.1534/g3.116.033050)
Supplement: Supplemental Material [file supp_g3.116.033050_FigureS3.pdf]

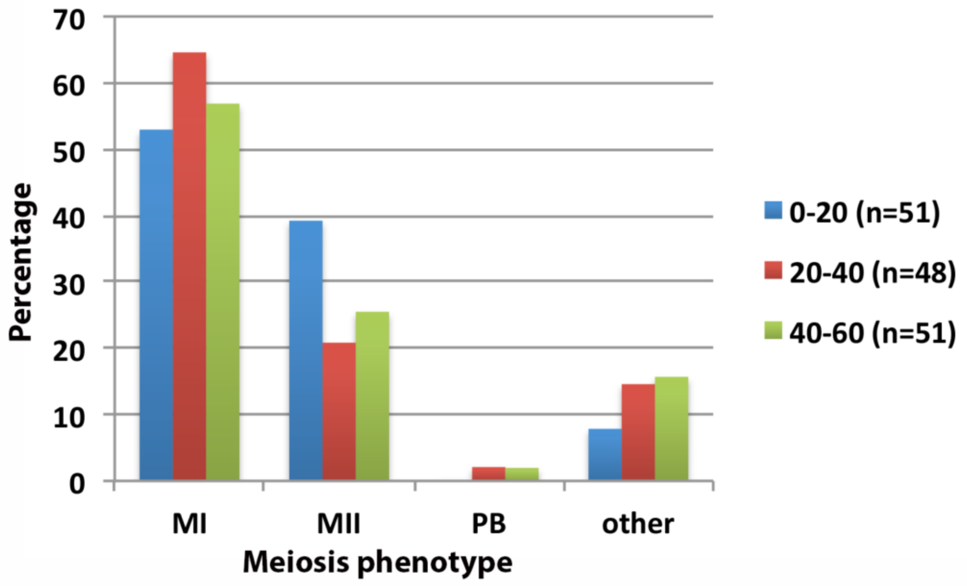

Figure S3

Meiosis phenotypes in timed egg collections from *CycB3*$^{L6/2}$. Eggs were collected at the indicated intervals and probed for DNA and Tubulin. Eggs were classified according to meiotic stage as MI (meiosis I), MII (meiosis II), PB (polar body), other (meiotic stage not able to be determined). Data comes from a single experiment with n values as indicated.
